# Mesenchymal Stromal/Stem Cells in Chronic Incomplete Traumatic Spinal Cord Injury: A Phase I/II Double-Blind Placebo-Controlled Multicentre Trial

**DOI:** 10.3390/biomedicines14040762

**Published:** 2026-03-26

**Authors:** Fernando Martins Braga, Hatice Kumru, Jesús Benito-Penalva, Joaquim Vives, Ruth Coll Bonet, Wanbao Ge, Luciano Rodríguez, Margarita Codinach, Aurora de la Iglesia-López, Antonio Gómez-Rodríguez, José Javier Cid-Fernández, Antonio Montoto-Marqués, Joan Vidal Samsó

**Affiliations:** 1 Spinal Cord Unit, Institut Guttmann Neurorehabilitation Hospital, 08916 Badalona, Barcelona, Spain; hkumru@guttmann.com (H.K.); jbenito@guttmann.com (J.B.-P.); 2 Institut de Neurociències (INC), Facultad de Medicina, Universitat Autònoma de Barcelona (UAB), 08193 Barcelona, Barcelona, Spain; 3 Germans Trias i Pujol Health Sciences Research Institute, 08916 Badalona, Barcelona, Spain; 4 Banc de Sang i Teixits (BST), 08005 Barcelona, Barcelona, Spain; jvives@bst.cat (J.V.); mcodinach@bst.cat (M.C.); 5 Vall d’Hebron Research Institute (VHIR), 08035 Barcelona, Barcelona, Spain; 6 Department of Medicine, Universitat Autònoma de Barcelona (UAB), 08035 Barcelona, Barcelona, Spain; 7 Spinal Cord Unit, University Hospital Complex of A Coruña (CHUAC), 15006 A Coruña, A Coruña, Spainantonio.montoto.marques@sergas.es (A.M.-M.); 8 Biomedical Research Institute of A Coruña, 15006 A Coruña, A Coruña, Spain

**Keywords:** spinal cord injuries, mesenchymal stem cells, allogeneic stem cell transplantation, regenerative medicine, neuroregeneration, neuroprotection, randomised controlled trial, electrophysiology, neuropathic pain, Wharton’s jelly-derived mesenchymal stem cells

## Abstract

**Background/Objectives:** Chronic traumatic spinal cord injury (SCI) causes persistent neurological deficits for which no clinically effective regenerative therapy is currently available. Mesenchymal stromal/stem cells (MSCs), particularly Wharton’s jelly-derived MSCs (WJ-MSCs), demonstrate immunomodulatory and neurotrophic potential. This phase I/II study evaluated the safety and efficacy of intrathecal allogeneic WJ-MSC administration in individuals with chronic incomplete cervical SCI. **Methods:** In this multicentre, randomised, double-blind, placebo-controlled trial (NCT05054803, EudraCT 2021-000346-18), 18 participants with chronic (1–5 years post-injury) incomplete cervical SCI (AIS B–D) received two intrathecal injections of WJ-MSCs (0.7–1.3 × 10^6^ viable cells/kg) or a placebo at baseline and 3 months. Seventeen participants completed the 12-month follow-up. Primary outcomes assessed safety, and secondary endpoints included International Standards for Neurological Classification of Spinal Cord Injury (ISNCSCI) motor and sensory scores, spasticity, neuropathic pain, functional independence, neurophysiological measures, and quality of life. **Results:** Intrathecal WJ-MSC administration was safe and well tolerated. Eighty adverse events occurred (placebo: 26; WJ-MSC: 54), predominantly mild or moderate; four severe events were unrelated to treatment. Both groups demonstrated significant within-group improvements in total motor scores at 12 months, with no between-group difference. No treatment effects were observed for sensory scores, electrophysiological measures, functional independence, spasticity, pain, or patient-reported outcomes. **Conclusions:** In this first randomised, placebo-controlled trial evaluating intrathecal WJ-MSCs in chronic incomplete cervical SCI, WJ-MSC administration demonstrated a favourable safety profile; however, no significant between-group differences were detected relative to the placebo. Given the limited sample size and early-phase design, the efficacy findings should be interpreted cautiously. Future research should explore enhanced cell products, intensified dosing schedules, optimised delivery strategies, early intervention, and multimodal therapeutic combinations.

## 1. Introduction

Traumatic spinal cord injury (SCI) affects approximately 26.5 cases per million each year worldwide, with cervical injuries accounting for most cases and carrying the highest burden of disability [1,2]. Most injuries are incomplete, and patients survive into the chronic phase, often facing lifelong motor, sensory, and autonomic deficits that severely impact their quality of life, create psychosocial burdens, and impose substantial economic costs on patients, caregivers, and healthcare systems [3,4,5].

Although acute-phase strategies focus on neuroprotection through rapid transfer, early surgery, and the optimisation of the spinal cord perfusion pressure, these measures cannot reverse established neurological damage once the injury has stabilised [6]. Despite advances in pharmacological and rehabilitative strategies, no approved standardised regenerative treatments have demonstrated the reliable and reproducible restoration of lost neurological function in the chronic phase [4,5,6].

Over the past two decades, stem cell-based interventions have emerged as one of the most actively explored regenerative strategies for SCI, with mesenchymal stromal/stem cells (MSCs) receiving the greatest clinical attention due to their availability, low immunogenicity, and multipotent reparative effects [6,7,8,9]. MSCs demonstrate multiple therapeutic mechanisms, including immunomodulation, neuroprotection, trophic support, remyelination, and the promotion of axonal plasticity, likely mediated through paracrine signalling pathways such as the BDNF-induced upregulation of GABAA receptor subunits and KCC2, which restore inhibitory neurotransmission and improve motor function [10,11,12,13].

Multiple early-phase trials suggest that MSCs—particularly those derived from bone marrow, adipose tissue, or the umbilical cord—exert anti-inflammatory and neurotrophic effects that may contribute to neural repair and the partial recovery of motor, sensory, or autonomic function, although robust evidence supporting direct axonal regeneration is lacking consensus [9,10]. A systematic review of 66 studies (*n* = 1086) reported functional improvements in patient subsets with predominantly mild, transient adverse events [14]. However, functional gains are mostly modest and vary widely between studies, with a recent meta-analysis indicating that, while approximately half of treated patients may improve by at least one American Spinal Injury Association (ASIA) Impairment Scale (AIS) grade, clinically meaningful functional restoration has not been consistently achieved across studies [9].

Wharton’s jelly-derived MSCs (WJ-MSCs) represent an attractive cell source, possessing a high proliferative capacity and favourable immunological properties and enabling non-invasive collection for standardised allogeneic use [10]. However, their efficacy in chronic SCI and optimal delivery protocols (route, dose, schedule) have not yet been optimally established [15,16].

The aim of this study was to evaluate the safety and tolerability of the intrathecal administration of allogeneic WJ-MSCs in patients with chronic incomplete cervical SCI and to assess secondary efficacy outcomes. This study constitutes, to our knowledge, the first randomised, placebo-controlled trial assessing WJ-MSC therapy in this specific population (chronic incomplete cervical SCI). The primary objective was to assess safety through physical examination, laboratory data, and adverse event monitoring up to 12 months after treatment initiation. Secondary objectives included evaluating changes from baseline in neurological, functional, sensory, pain, spasticity, quality of life, and psychological measures at predefined time points.

## 2. Materials and Methods

### 2.1. Study Design and Participants

This phase I/II, multicentre, randomised, double-blind, placebo-controlled trial (ClinicalTrials.gov NCT05054803, EudraCT 2021-000346-18) evaluated the safety of two intrathecal infusions of allogeneic WJ-MSCs in patients with chronic (1–5 years post-injury) traumatic SCI.

The study was approved by the Spanish Agency of Medicines and Medical Devices (AEMPS) and the local Clinical Research Ethics Committee (Fundació Unió Catalana d’Hospitals; protocol number 21–36). All procedures complied with the Declaration of Helsinki and Good Clinical Practice guidelines.

Selection criteria are provided in Table 1.

The sample size was planned for an early-phase safety evaluation and feasibility of trial procedures; no a priori statistical power calculation was performed for secondary efficacy endpoints.

Allocation was implemented using a 1:1 randomisation sequence generated and held by the study sponsor/cell provider (Banc de Sang i Teixits), which had no role in participant recruitment, clinical care, outcome assessment, or rehabilitation delivery. Participants were enrolled at the study sites by local investigators and assigned sequentially to the next available study code; treatment kits were provided prefilled and labelled with the corresponding code. Allocation information was concealed from participants and all site personnel involved in patient contact, clinical management, and outcome assessments. No stratification by centre or AIS grade was applied.

**Figure 1:**
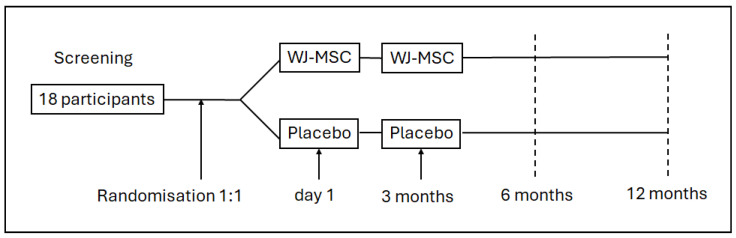
Study design. WJ-MSC: group receiving cell treatment.

**Figure 2:**
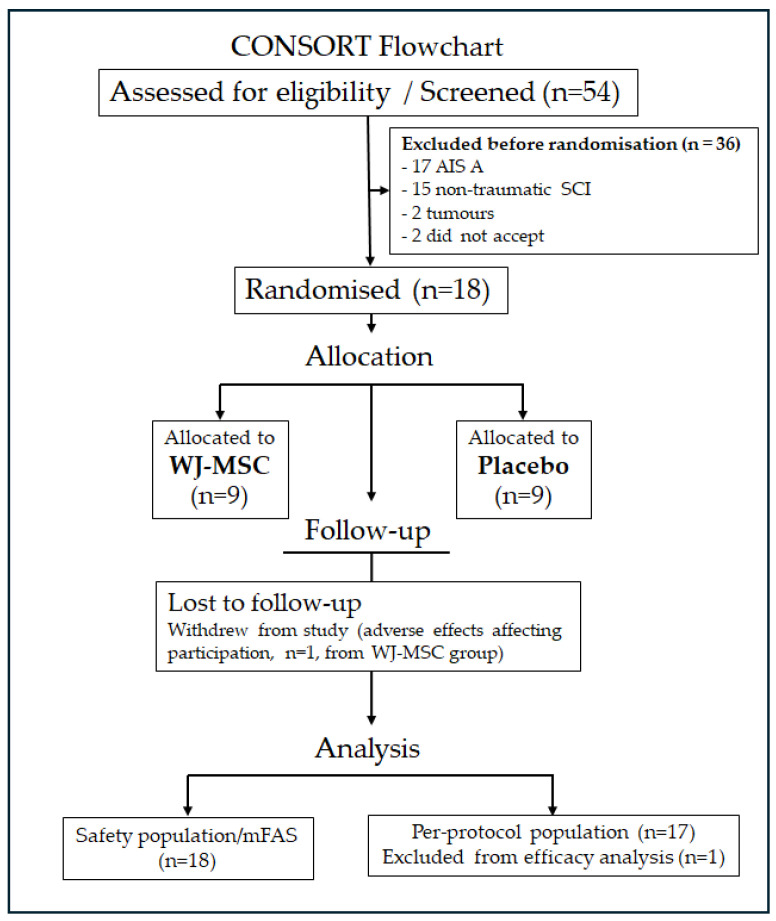
Flowchart.

The study design is shown in Figure 1, and the CONSORT flowchart is presented in Figure 2. Of the 54 individuals screened, 36 were excluded prior to randomisation (17 AIS A; 15 non-traumatic SCI; 2 tumours; 2 declined participation), as shown in Figure 2. Eighteen participants were enrolled and randomised in a 1:1 ratio to the WJ-MSC (*n* = 9) or placebo (*n* = 9) group.

### 2.2. Study Protocol

Participants underwent evaluations at screening (pre-injection), baseline (first injection), and all follow-up visits, including a second injection at 3 months (Table 2). All participants followed a rehabilitation programme, individualised according to lesion characteristics and functional needs. Rehabilitation was delivered over approximately 6–8 months. Concomitant medications were continued as per each participant’s usual care; no protocol-mandated medication changes were made for study purposes, and relevant concomitant medications were recorded in the data collection form and medical record at each visit. Blood and CSF samples were collected and cryopreserved for future exploratory miRNA biomarker analyses, which are outside the scope of the present report. Follow-up continued for 12 months after the baseline assessment.

Recruitment occurred between October 2021 and June 2022, with the final participant completing follow-up in June 2023. Written informed consent was obtained from all participants before enrolment. No data from this cohort have been previously reported. Participants did not receive financial compensation. The trial was conducted at the Spinal Cord Unit of Institut Guttmann Neurorehabilitation Hospital (Badalona, Catalonia, Spain) and the Spinal Cord Unit of University Hospital Complex of A Coruña (A Coruña, Galicia, Spain).

The preparation of this manuscript followed the CONSORT 2025 statement; the completed CONSORT 2025 checklist is provided in the Supplementary Materials (Table S1).

### 2.3. Interventions

Participants received two intrathecal administrations: at baseline (first injection) and at 3 months (second injection) (Table 2). The investigational product consisted of WJ-MSCs supplied by Banc de Sang i Teixits in prefilled, blinded syringes labelled with a study code. The final product was prepared as a suspension corresponding to 0.7–1.3 × 10^6^ viable cells/kg, as specified in the study protocol. The dose range and two-administration schedule (baseline and 3 months) were selected based on prior clinical experience with intrathecal WJ-MSC administration at a similar dose range [17,18,19] and to allow an interval for safety assessment while exploring whether a second administration could help reinforce any short-lived paracrine effects.

Due to the blinded supply of centrally prepared, coded, prefilled syringes, total viable cell counts per administered syringe were not available to investigators; dosing is therefore reported as the protocol-specified range.

The placebo consisted of sterile saline solution Plasmalyte 148 (Baxter, Deerfield, IL, USA) supplemented with 2% (*w*/*v*) human serum albumin (Albuplan, Grifols (Sant Cugat del Vallès, Spain)), identical in volume, appearance, and labelling to the prefilled WJ-MSC syringes to ensure indistinguishability.

Intrathecal administration was performed by lumbar puncture at the L3–L4 interspace under aseptic conditions. Immediately before administration, 4 ± 1 mL of cerebrospinal fluid (CSF) was collected. The assigned study treatment (WJ-MSCs or placebo) was then administered intrathecally as a total syringe volume of 4 ± 1 mL over approximately 2–5 min. After the procedure, participants remained supine and under observation for at least 2–4 h, with clinical monitoring for adverse events. Blinding success was not assessed.

### 2.4. WJ-MSC Collection, Preparation, and Administration

WJ-MSCs were derived, expanded, and characterised under Good Manufacturing Practice (GMP)-compliant conditions and following informed donor consent [20,21].

Briefly, umbilical cord fragments were dissected longitudinally, and Wharton’s jelly was mechanically extracted and plated onto the surfaces of T-flasks (TPP, Trasadingen, Switzerland) with reclosable lids. The tissue was incubated at 37 °C for 30 min before adding Dulbecco’s modified Eagle medium (DMEM; Gibco, Carlsbad, CA, USA) supplemented with 10–20% human serum (Banc de Sang i Teixits, Barcelona, Spain), 20 mg/mL streptomycin, and 120 µg/mL amphotericin B (Invitrogen, Carlsbad, CA, USA). After 2–5 days, adherent cells were washed with saline and cultured in antibiotic-free medium, replaced every 3–4 days, until reaching a density of 1–3 × 10^3^ viable cells/cm^2^.

A master cell bank (MCB) was cryopreserved at ≥5 × 10^6^ viable cells. Upon thawing, further expansion produced the drug product (DP) cryovials. The final product (FP), defined as a suspension of 0.7–1.3 × 10^6^ viable cells/kg, was prepared by thawing, washing, and resuspending the DP in saline for intrathecal administration.

Cell identity was confirmed by flow cytometry (CD45^−^, CD105^+^, CD73^+^, CD31^−^, HLA-DR^−^), and immunomodulatory potency was demonstrated by ≥30% inhibition of lymphocyte proliferation in validated co-culture assays, whose design has been comprehensively reported elsewhere [22]. All batches met predefined release criteria, including sterility (absence of bacterial, fungal, or Mycoplasma contamination), absence of adventitious viruses, and endotoxin levels < 1.00 EU/mL (Table 3), consistent with the validation of the manufacturing process reported elsewhere [20,21].

### 2.5. Immunopotency Assay

The immunomodulation potential of WJ-MSCs was determined by their capacity to inhibit the proliferation of polyclonally stimulated lymphocytes in vitro, following an improved method based on that originally described by Oliver-Vila et al. [20], as described next. Briefly, 2.5 × 10^6^ PBMCs/mL were labelled with 0.625 μM carboxy-fluorescein diacetate succinimidyl ester (CFSE) for 10 min using the CellTrace™ CFSE Cell Proliferation Kit (Molecular Probes, Eugene, OR, USA). After washing, (1–2) × 10^7^ cells/mL were incubated for 12 min at 37 °C, washed again, and seeded onto flat-bottomed 24-well plates (Corning, Corning, NY, USA) at a 5:1 peripheral blood mononuclear cell (PBMC)–WJ-MSC ratio. Lymphocytes were activated with 25 ng/mL phorbol 12-myristate 13-acetate (PMA, Sigma-Aldrich (St. Louis, MO, USA)) and 0.5 μM ionomycin (Sigma-Aldrich) in a final volume of 0.5 mL/well of DMEM containing 2 mM glutamine and supplemented with 10% hSerB. The proliferation of PBMCs was determined by measuring the reduction in the fluorescence intensity at day 5 by flow cytometry using a Navios EX (Brea, CA, USA), and raw data were analysed with Kaluza Analysis Software (version 2.1, Beckman Coulter (Brea, CA, USA)). Inhibition of PBMC proliferation was calculated using Equations (1)–(3):

(1) $$Absolute\;proliferation=\bar{stimulated}-\bar{nonstimulated}$$

(2) $$Normalised\;proliferation\left(\%\right)=\frac{absolute\;proliferation\;with\;MSC}{absolute\;proliferation\;without\;MSC}\times 100$$

Inhibition (%) = 100 − normalised proliferation of co-culture(3)

### 2.6. Clinical Evaluation

Neurological status was assessed using the International Standards for Neurological Classification of Spinal Cord Injury (ISNCSCI) to determine the motor and sensory scores, neurological level, and overall injury severity (ASIA Impairment Scale) [23]. Peripheral nerve involvement was excluded through standard clinical examination. Lower-limb spasticity at the hip and knee was evaluated with the Modified Ashworth Scale (MAS) [24], and hand muscle strength was quantified using a wireless handgrip dynamometer and pinchmeter (Biometric E-Link version 16, Newport, UK), assessing cylindrical grip, lateral pinch, and tip-to-tip pinch, with three trials performed per hand. Neuropathic pain at and below the injury level was rated on an 11-point numerical rating scale (NRS; 0 = no pain, 10 = worst imaginable pain) [25]. Functional independence was measured with the Spinal Cord Independence Measure III (SCIM III) [26] and supplemented by the Walking Index for Spinal Cord Injury II (WISCI II) to evaluate ambulation capacity [27]. Health-related quality of life was assessed with the World Health Organization Quality of Life—BREF (WHOQOL-BREF) questionnaire [28]. Psychological status was evaluated using the Hospital Anxiety and Depression Scale (HADS) [29] and the Psychological Well-Being Index (PWI) [30]. Finally, community integration was examined with the Community Integration Questionnaire—Institutional Version (CIQ-IG) [31].

### 2.7. Neurophysiological Assessment

Neurophysiological testing included motor and somatosensory evoked potentials, reflex studies (F-wave and H-waves), and quantitative sensory testing (QST). All procedures were performed bilaterally at baseline and at 12 months after the first infusion using a Synergy EMG/EP system (Oxford Instruments, High Wycombe, UK) by a trained neurophysiologist, following standardised acquisition protocols [32].

Transcranial magnetic stimulation (TMS) was applied over the vertex corresponding to the primary motor cortex using a Magstim Super Rapid stimulator (Magstim Company, Whitland, UK) equipped with a double-cone coil. Single magnetic pulses were delivered to obtain motor evoked potentials (MEPs) in the tibialis anterior (TA), abductor hallucis (AH), and abductor digiti minimi (ADM) muscles. Four trials per muscle were recorded bilaterally. Signals were amplified (0.1 mV/div), band-pass-filtered (10 Hz–10 kHz), and visually inspected for reproducible MEPs. Latency and peak-to-peak amplitude were measured for each muscle.

Somatosensory evoked potentials (SSEPs) were recorded by electrical stimulation of the posterior tibial nerve at the retromalleolar region using intradermal needle electrodes (pulse duration 1 ms, repetition rate 3 Hz, maximum intensity ≤ 30 mA). Cortical responses were recorded at Cz–Fz with a 100 ms analysis window, amplification of 1 mV/div, and band-pass filtering between 3 Hz and 3000 Hz. Two averaged responses of 300 sweeps per side were obtained to confirm reproducibility. The latency and amplitude of the cortical components were measured for analysis.

F-waves were recorded bilaterally from the abductor hallucis (AH) and abductor digiti minimi (ADM) muscles following supramaximal stimulation of the corresponding motor nerves at 0.5 Hz. Twenty stimuli were delivered, and the minimal latency, amplitude, and persistence were calculated.

H-reflexes were obtained from the soleus muscle by stimulation of the posterior tibial nerve in the popliteal fossa. The peak-to-peak amplitude and H/M ratio were measured. Both tests were used to assess the excitability and conduction of segmental reflex pathways. As for QST, the electrical perception threshold (EPT) and electrical pain perception threshold (EPPT) were determined bilaterally at seven dermatomes per participant: one dermatome rostral to the neurological level of injury (considered as “level 0”) and six caudal dermatomes (−1, −2, −5, −7, −9 and −12). Electrical square-wave stimuli (0.5 ms duration, 3 Hz rate) were delivered to the key AIS sensory points using the above-mentioned Synergy system. The stimulation intensity was gradually increased until participants reported the first perception (EPT) and the first painful sensation (EPPT). To prevent current spread, the maximum stimulus intensity was limited to 40 mA. Each measurement was repeated three times, and mean values were used for analysis.

### 2.8. Safety Indicators and Efficacy Endpoints

The primary safety assessment was descriptive and based on predefined indicators: the incidence and severity of adverse events (including serious adverse events and events considered related to study treatment), changes in vital signs, and clinically relevant laboratory abnormalities over the 12-month follow-up. Adverse events were classified by severity (mild, moderate, severe) and seriousness (serious/SAE vs. non-serious), assessed for relatedness (related to study procedures, potentially related to study treatment, or unrelated), and coded using the Medical Dictionary for Regulatory Activities (MedDRA) System Organ Class (SOC).

The key secondary efficacy endpoints were changes from baseline in ISNCSCI neurological scores (total motor score, light touch, and pin prick). Additional secondary efficacy endpoints included spasticity (MAS), neuropathic pain (NRS), functional independence (SCIM III and WISCI II), electrophysiological measures (MEPs, SSEPs), quantitative sensory thresholds (EPT/EPPT), quality-of-life indices (WHOQOL-BREF), psychological outcomes (HADS, PWI), and community integration scores (CIQ-IG). These secondary endpoints were chosen to capture both objective functional changes and patient-reported outcomes within the intended scope of this trial.

### 2.9. Statistical Analysis

The safety population included all participants who received at least one intrathecal infusion. The efficacy population comprised all participants who completed the 12-month follow-up; one participant who was withdrawn after the first infusion was excluded from the efficacy analyses but retained for safety evaluations.

Baseline demographic and clinical characteristics were summarised using means and standard deviations (SDs) or medians with interquartile ranges (IQRs, Q1–Q3) for continuous variables and with frequencies and percentages for categorical variables. For the total motor, light touch, and pin prick ISNCSCI scores, within-group changes from baseline to 12 months were assessed using paired *t*-tests. Between-group differences in change scores were compared using independent-samples *t*-tests, with Mann–Whitney U tests performed as non-parametric sensitivity analyses. Results are presented as the mean ± SD, median (Q1–Q3), or proportions, as appropriate. All statistical tests were two-sided, with a significance threshold of *p* < 0.05.

Longitudinal secondary outcomes—including functional measures (SCIM III, WISCI II), grip strength parameters, quantitative sensory testing variables (EPT, EPPT), electrophysiological measures (MEP, SSEP, F-wave, H-reflex), spasticity, pain, quality-of-life scores, and psychological and community integration assessments—were analysed using linear mixed-effects models estimated using restricted maximum likelihood (REML), with fixed effects for the treatment group, time, and group-by-time interaction and participant-specific random intercepts to account for within-participant correlation. Model assumptions were evaluated through standard diagnostic procedures.

Analyses were performed using complete cases, and missing data resulting from the withdrawal of one participant were not imputed. A sensitivity analysis including all randomised participants (intention-to-treat) was conducted to confirm the robustness of the findings. No adjustments were made for multiple comparisons, given the prespecified secondary nature of these analyses; these analyses should be considered exploratory. All analyses were performed using IBM SPSS Statistics for Windows, version 27.0 (IBM Corp., Armonk, NY, USA).

## 3. Results

### 3.1. Study Population

**Figure 3:**
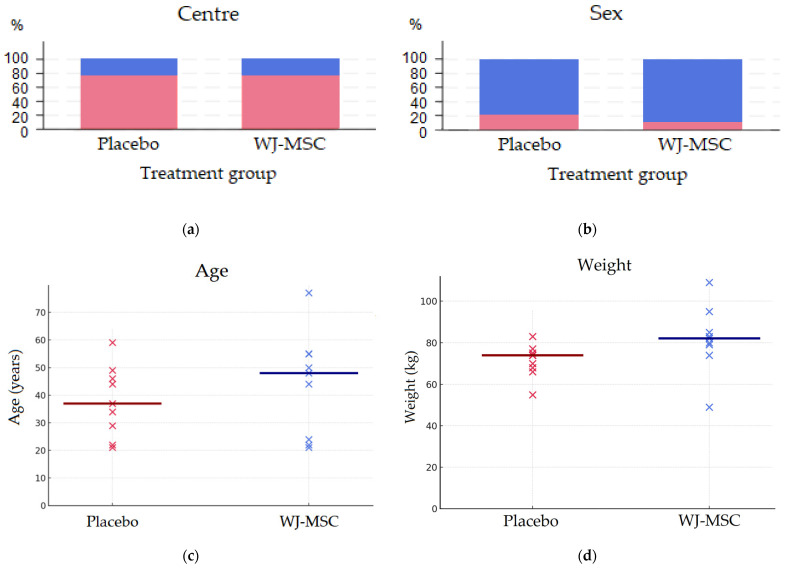
Demographic distribution of participants in Wharton jelly mesenchymal stem cell (WJ-MSC) versus placebo group. (**a**) Centre: blue—University Hospital Complex of A Coruña (CHUAC), red—Institut Guttmann Neurorehabilitation Hospital (IG). (**b**) Sex: blue—male, red—female. (**c**) Participant’s age (in years): red—placebo, blue—WJ-MSC. (**d**) Participant’s weight (in kilograms): red—placebo, blue—WJ-MSC.

A total of 54 individuals were screened, of whom 18 were enrolled and randomised 1:1 to receive either intrathecal WJ-MSCs (*n* = 9) or a placebo (*n* = 9). Baseline demographics and injury characteristics are summarised in Table 4. The mean age was 41 years (range 21–77), and most participants were male (placebo, 77.8%; WJ-MSCs, 88.9%). The mean time since injury was 2.3 years (range: 1.1–3.3) in the placebo group and 2.8 years (range: 1.1–4.3) in the WJ-MSC group. The distribution of participants according to the centre (IG vs. CHUAC), sex, age, and weight is shown in Figure 3.

At baseline, the placebo group included four participants classified as AIS B, three as AIS C, and two as AIS D; the WJ-MSC group included two AIS B, four AIS C, and three AIS D. The mean baseline total motor score was 44 (median: 38, range: 10–89) in the placebo group and 47.4 (median: 45, range: 20–76) in the WJ-MSC group (Table 4 and Table 5).

One participant allocated to the WJ-MSC group was found to be 77 years old, exceeding the upper age limit specified in the inclusion criteria. This participant was withdrawn early due to intolerance to motor evoked potential testing and the subsequent identification of the age criterion violation. Because the first dose of cell treatment had already been administered, this participant was included in the safety analysis but excluded from efficacy analyses. All other 17 participants completed the 12-month follow-up as per protocol.

### 3.2. Safety Outcomes

A total of 80 adverse events (AEs) were recorded: 26 in the placebo group and 54 in the WJ-MSC group. Most AEs were mild (52/80, 65%), affecting 14 participants overall. Moderate AEs accounted for 24 events (30%), occurring in 13 participants (five in the placebo group and eight in the WJ-MSC group). Four events (5%) were classified as severe and affected three participants in total: one placebo participant experienced a urinary tract infection, while one pressure ulcer, one respiratory infection, and one urinary tract infection affected two participants in the WJ-MSC group. However, none of the severe AEs were considered related to the study treatment. No life-threatening or unexpected AEs occurred. Notably, immunological monitoring of CSF revealed no de novo anti-HLA antibodies or persistent donor DNA, consistent with the absence of immune-mediated AEs in all treated participants.

The most frequent AE was headache attributed to lumbar puncture, reported in six of nine participants in the placebo group and seven of nine in the WJ-MSC group. These headaches were transient and self-limiting. Other AEs, such as musculoskeletal pain, spasms, or urinary tract infections, were assessed individually for relatedness and were not considered related to the study procedures or study treatment. Table 6 summarises the relevant AEs per participant over the 12-month follow-up; “relevant” referred to those AEs considered related to study procedures or potentially related to treatment; all events in the table fell within one of these categories. Mild, moderate, or severe AEs unrelated to study procedures or study treatment are not included in Table 6 but are included in the overall AE counts reported above and summarised in Supplementary Table S2. Vital sign changes were observed in association with reported adverse events (including autonomic dysreflexia), with no clinically relevant laboratory abnormalities during follow-up.

### 3.3. Neurological Outcomes

Both the placebo and WJ-MSC groups demonstrated significant within-group improvements in total motor scores from baseline to 12 months, as shown by the paired *t*-tests (both *p* < 0.05) (Table 7). However, an intergroup comparison of the change scores using an independent-samples *t*-test, supported by a Mann–Whitney U test, showed no significant differences between the two groups (both *p* > 0.6).

For light touch total scores, neither the placebo group nor the WJ-MSC group showed significant within-group changes over 12 months (paired *t*-tests: *p* = 0.152 and *p* = 0.116, respectively). The between-group comparison of the light touch change scores demonstrated no significant differences between the placebo and WJ-MSC groups, based on both the independent-samples *t*-test and the Mann–Whitney U test (both *p* > 0.2). For pin prick total scores, neither the placebo group nor the WJ-MSC group demonstrated significant within-group changes from baseline to 12 months, as shown by the paired *t*-tests (both *p* > 0.1). Furthermore, the between-group comparison of the pin prick scores revealed no significant differences between treatments, according to both the independent-samples *t*-test and the Mann–Whitney U test (both *p* > 0.1).

One participant in the placebo group improved by at least one AIS grade (C to D), as shown in Table 4; none improved in the WJ-MSC group. A sensitivity analysis including all randomised patients showed similar effect estimates and *p*-values.

### 3.4. Electrophysiological Outcomes

**Figure 4:**
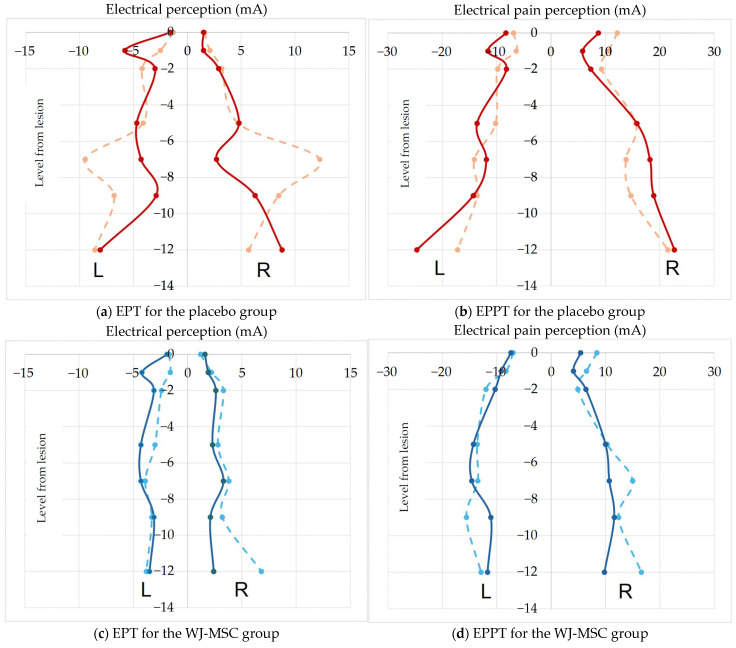
Electrical perception thresholds (EPTs, first perception) and electrical pain perception thresholds (EPPTs, painful sensation) at baseline and 12 months, red—placebo, blue—WJ-MSC. (**a**) EPT for the placebo group. (**b**) EPPT for the placebo group. (**c**) EPT for the WJ-MSC group. (**d**) EPPT for the WJ-MSC group. Dashed line: baseline; solid line: 12 months. L/R: left/right. *X*-axis: current intensity (milliamperes, mA). *Y*-axis: dermatomal level (at lesion level: 0; below the lesion level: −1, −2, −5, −7, −9, −12) relative to the neurological level of injury.

Quantitative sensory testing demonstrated no treatment effect. EPTs and EPPTs were measured bilaterally in 14 dermatomes per participant. Trajectories for both thresholds were comparable in the WJ-MSC and placebo groups (Figure 4), and mixed-effects analyses revealed no significant treatment-by-time interactions.

Motor and somatosensory evoked potentials remained stable during follow-up. The motor evoked potential latency to the right abductor digiti minimi muscle decreased modestly from approximately 45 ms at baseline to 38–40 ms at 12 months in both groups, but this change did not differ between WJ-MSC and placebo recipients (*p* = 0.450). MEP and SSEP latencies and amplitudes showed no significant improvement attributable to WJ-MSCs (Tables S3–S7).

The latencies and amplitudes of the M-max and F-waves, as well as F-wave persistence and the Fmax/Mmax ratio, measured bilaterally in the upper extremities, together with the H-wave and Hmax/Mmax ratio in the lower extremities, did not show any significant changes in either group. The evolution of these parameters was comparable in both groups, with no significant treatment-by-time interaction detected (all *p* > 0.05).

In summary, the MEPs and SSEPs in the upper and lower extremities, the F- and H-wave assessments, QST, and spinal cord excitability measured by EPT and EPPT revealed no differences between treatment groups.

### 3.5. Functional Measures

Spasticity scores on the MAS remained stable throughout the study in both treatment groups. Neuropathic pain ratings decreased slightly over time in both groups (mean scores ranged from approximately 2.9 to 1.9 on the 0–10 scale), but the treatment effect was not significant (*p* = 0.425).

Functional independence scores (SCIM III) increased slightly over the 12-month follow-up in both treatment groups. A mixed-effects analysis confirmed no significant treatment effect, with estimated differences of –2.52 points (95% CI: –23.1 to 18.0) at month 6 and 2.16 points (95% CI: –0.80 to 6.08) at month 12 (*p* = 0.810 and *p* = 0.130, respectively).

With regard to the grip strength assessments, at baseline, participants demonstrated substantial motor impairment in the cylindrical grip, spherical grip, and lateral pinch tests, with median values near zero, because many participants were unable to exert a measurable force. Over the 12-month follow-up period, slight improvements in grip strength were observed in both treatment groups in all three configurations. However, these changes were comparable between groups, and no treatment effect or treatment-by-time interaction was detected in any assessment (all *p* > 0.05). Mixed-effects models consistently resulted in between-group estimates close to zero with wide confidence intervals. Variability in grip performance, as measured by the coefficient of variation, decreased over time in both groups, suggesting improved consistency with repeated testing rather than a treatment-specific effect. In summary, grip strength assessments revealed no differential benefit attributable to WJ-MSC treatment, with the observed improvements likely reflecting natural recovery, rehabilitation effects, or enhanced test familiarity.

### 3.6. Patient-Reported Outcomes

Patient-reported outcomes showed no statistically significant differences between treatment groups. Neuropathic pain levels were low at baseline and remained low throughout follow-up. At baseline, the median score on the 0–10 numeric pain scale was 2.7 in the placebo group and 0.9 in the WJ-MSC group. By 12 months, both groups had a median pain score of 0 (no pain). There was a slight decrease in average pain in the placebo group (mean: 2.9 → 1.9 from baseline to 12 months), but this trend did not reach significance (treatment-by-time *p* = 0.470), while the WJ-MSC group remained roughly unchanged (2.1 → 2.2).

Quality of life as measured by the WHOQOL-BREF questionnaire did not significantly change or differ between treatment groups. Both groups reported stable quality-of-life scores over time, with no treatment-related enhancement in physical or psychological domains.

Psychological and social outcomes were likewise comparable. The HADS anxiety and depression scores remained in the mild range on average and showed no meaningful changes from baseline in either group (*p* > 0.5). The PWI scores and the CIQ-IG results indicated that both groups maintained comparable psychosocial statuses during follow-up. There was no evidence of a greater improvement in mood or social integration with WJ-MSC treatment. Patients in both groups typically reported stable or slight improvements in subjective well-being.

## 4. Discussion

This phase I/II trial provides the first controlled evidence regarding the intrathecal administration of allogeneic WJ-MSCs in chronic incomplete cervical SCI. In this multicentre, randomised, double-blind, placebo-controlled study, two intrathecal doses of WJ-MSCs were safe and generally well tolerated, with no major adverse effects attributable to the cell product. However, despite this favourable safety profile, the intervention did not result in superior neurological or functional outcomes compared with the placebo.

### 4.1. Safety Profile

Our safety findings align with prior trials of intrathecal MSC administration in SCI. A recent phase I study delivered allogeneic WJ-MSCs via multiple routes (intrathecal, intramuscular, and intravenous) in chronic SCI. No serious treatment-related adverse effects occurred, with only transient mild events, including subfebrile fever in four patients, moderate post-injection headaches in two, and self-limited injection-site soreness in two [15]. Similarly, a randomised controlled trial (RCT) by Albu et al. (2020) using intrathecal WJ-MSCs versus a placebo in complete SCI patients showed no significant side effects except mild post-lumbar puncture headache with brief vomiting in one patient and transient injection-site pain in another [17]. Vaquero et al. demonstrated that even repeated high-dose intrathecal autologous bone marrow-derived MSCs (three 100-million-cell injections) were well tolerated, with no adverse events attributable to the cell treatment [33].

In these previous studies, as well as in the present trial, adverse events were predominantly mild or moderate and transient. Most were procedure-related (e.g., temporary post-puncture headache or local injection trauma) rather than cell-related. To our knowledge, no trials have reported serious complications such as infections, neurological deterioration, or immune-mediated reactions following WJ-MSC therapy. This favourable safety profile is consistent with the low immunogenicity of WJ-MSCs, which express minimal HLA-DR and can be transplanted allogenically without provoking rejection [34]. These data strongly support the safety of intrathecal WJ-MSC administration in chronic SCI.

### 4.2. Efficacy Outcomes

Concerning efficacy outcomes, WJ-MSC administration produced within-group changes—most notably, a significant improvement in total motor scores over 12 months. However, comparable gains were observed in the placebo group, resulting in no between-group differences. Sensory outcomes likewise failed to demonstrate a treatment effect: neither the light touch nor pin prick scores showed significant within-group changes in either group; between-group comparisons revealed no significant differences. AIS grade conversion occurred in one participant in the placebo group and in none of the WJ-MSC recipients. Overall, these findings do not demonstrate the superiority of WJ-MSC treatment across neurological, functional, electrophysiological, or patient-reported outcomes. Given the multiple exploratory endpoints and relatively small sample size, isolated within-group findings should be interpreted cautiously as hypothesis-generating rather than confirmatory.

Our results highlight the importance of including a control group in chronic SCI trials. Spontaneous fluctuations, rehabilitation effects, regression to the mean, and measurement variability (including potential test–retest learning) can create the appearance of an improvement even in the absence of active therapy and may have contributed to the within-group improvements observed in both groups. Without a controlled comparison, such intragroup changes could easily be misinterpreted as evidence of efficacy.

RCTs using intrathecal MSCs in chronic SCI, however, are rare. Awidi et al. compared perilesional and intrathecal autologous bone marrow MSCs against intrathecal allogeneic umbilical cord MSCs in 20 chronic SCI patients. This study reported improvements from baseline in both groups; motor gains were greater in the surgical (perilesional, followed by intrathecal administration) group. However, the open-label design and absence of a placebo control limit causal inference, although the results suggest that the dosing intensity and delivery to the lesion may matter [35].

Single-patient studies and phase I trials further suggest feasibility and safety but do not conclusively establish the efficacy of MSCs. A case report of repeated high-dose intrathecal WJ-MSCs in complete chronic SCI by Jamali et al. described AIS grade conversion with improvements in motor, sensory, bladder, and daily function over 25 months, without serious toxicity [36]. A single-arm trial on intrathecal autologous adipose-derived MSCs by Bydon et al. (*n* = 10) reported no serious adverse events and found AIS grade improvements in 7/10 participants, but the authors cautioned that the lack of controls made it impossible to determine whether the changes were due to the cell treatment [37].

Another factor that may contribute to differences across MSC studies in chronic SCI is the dosing regimen. In the present trial, participants received two intrathecal administrations delivered 3 months apart. Other trials have used single-dose protocols, such as Bydon et al. [37]; monthly three-dose schedules, such as Awidi et al. [35]; or more intensive repeated intrathecal administration, as in the single-patient case report by Jamali et al. [36]. Such differences in dosing frequency and cumulative exposure may influence the observed efficacy outcomes, although direct comparison is limited by variations in the MSC source, delivery approach, injury characteristics, and study design. These studies are encouraging, but the evidence is anecdotal rather than definitive.

Previous non-placebo-controlled studies with intramedullary MSC injection have reported functional outcomes favouring MSC-treated groups over controls, with Cheng et al. describing gains relative to rehabilitation alone [18] and Dai et al. showing improvements compared with untreated controls [38]. Similarly, El-Kheir et al. conducted a single-blind controlled study in which the intrathecal administration of autologous adherent bone marrow MSCs combined with physical therapy was associated with greater functional improvements than physical therapy alone [39].

Several biological factors may account for the absence of a detectable treatment effect. Chronic SCI is characterised by extensive gliosis, cystic cavitation, and a hostile extracellular microenvironment that presents substantial barriers to cellular integration, axonal regeneration, and trophic support. MSCs exert their therapeutic effects primarily through paracrine signalling—via neurotrophic, angiogenic, and immunomodulatory factors—rather than through direct cellular replacement. These paracrine effects are inherently short-lived and may be limited in their ability to modify the stable, inhibitory microenvironment in long-standing injuries. Moreover, intrathecal administration relies on passive cerebrospinal fluid distribution, which may limit parenchymal penetration and cell–tissue interactions at the lesion site, particularly in the presence of established glial scarring.

### 4.3. Limitations

The relatively small sample size of the current study limited the statistical power to detect treatment effects, particularly given the heterogeneity of incomplete cervical injuries. As an early-phase trial primarily designed for safety evaluation, the study was not powered for definitive efficacy testing. The 12-month follow-up may have been insufficient to capture delayed regenerative effects. Intrathecal delivery, although minimally invasive, may not optimise cell retention at the injury site compared to direct intralesional/perilesional methods. The two-dose regimen represents a conservative approach; more frequent administration may be required to maintain paracrine signalling. Rehabilitation was individualised; therefore, variations in rehabilitation programmes and other non-specific factors (e.g., test–retest effects) may have contributed to the within-group improvements in the placebo and WJ-MSC groups alike. Finally, our broad inclusion criteria may have reduced the ability to identify treatment effects that could be more detectable in specific patient subgroups with greater neuroplastic potential. In addition, multiple secondary endpoints were assessed without adjustment for multiplicity, and the findings should therefore be interpreted with appropriate caution.

### 4.4. Implications for Future Research

Three key implications emerge for future trial design. Firstly, uncontrolled case series suggest that more intensive or repeated intrathecal dosing may be necessary to achieve a therapeutic benefit. The “100/3” guideline (three intrathecal doses of approximately 100 × 10^6^ MSCs) has proven both feasible and safe in chronic spinal cord injury, supporting the exploration of more frequent dosing schedules within controlled study frameworks [33]. Secondly, multimodal approaches that combine cell therapy with structural or surgical lesion targeting merit investigation. Strategies employing intralesional delivery or the use of scaffolds have reported sensory and functional improvements in chronic complete spinal cord injury with acceptable tolerability, justifying trials that integrate lesion-targeted components with intrathecal Wharton’s jelly-derived MSCs [35,40]. Thirdly, refining patient selection and outcome measures may improve the detection of potential treatment effects. Increasing trial populations for subgroups with greater neuroplastic potential—such as individuals with more residual tissue bridges or shorter injury chronicity—and incorporating sensitive, objective biomarkers, including diffusion MRI, could substantially increase assay sensitivity [37].

## 5. Conclusions

The intrathecal administration of allogeneic WJ-MSCs was feasible and generally well tolerated in this early-phase randomised trial on chronic, incomplete cervical SCI. No significant between-group differences in neurological, functional, or patient-reported outcomes were observed at 12 months, despite within-group improvements in both the treatment and placebo groups. Although open-label and single-arm studies have suggested potential benefits of MSC therapies, our findings do not provide evidence supporting the superiority of intrathecal MSC monotherapy delivered in a two-dose regimen over a placebo. Given the limited sample size, the secondary nature of the efficacy analyses, and the limited power to support definitive conclusions regarding efficacy, these results should be interpreted with caution. Future research should investigate whether enhanced or primed MSC products (e.g., preconditioned, genetically modified, or encapsulated cells), earlier intervention, or multimodal strategies that integrate cell therapy with rehabilitation protocols, biomaterial scaffolds, lesion-targeted delivery, or combined neuromodulation may improve the therapeutic response.

## Figures and Tables

**Table 1:** Selection criteria.

**Inclusion criteria**
Traumatic spinal cord injury involving cervical segments C1–T1, confirmed by MRI
Incomplete injury (ASIA Impairment Scale grade B, C, or D)
Time since injury 1–5 years
Age 18 to 70 years
Participant residing close to the study centre and able to attend follow-up visits
Written informed consent and ability to understand study requirements
**Exclusion criteria**
Dependence on mechanical ventilation
Pregnancy or breastfeeding
Women of childbearing potential without effective contraception
Planned spinal surgery within 12 months of enrolment
Neurodegenerative disease or recent (<5 years) or active malignancy
Significant laboratory abnormalities that contraindicate participation
Communication barriers that preclude assessment
Recent (<30 days) experimental treatment that could interfere with results
Prior treatment with an advanced therapy medicinal product (e.g., cell therapy)
Contraindication to lumbar puncture or to participation in a rehabilitation programme
Other conditions or circumstances that may compromise study participation

**Table 2:** Timeline of the study.

Procedure	Baseline	V1 (t = 0)	V2 (1 w)	V3 (1 mo)	V4 (3 mo)	V5 (V4 + 1w)	V6 (4 mo)	V7 (6 mo)	V8 (9 mo)	V9 (12 mo)
ASIA neurological examination	X			X	X		X	X		X
Physical examination	X				X			X		X
Vital signs check	X	X			X					X
Blood tests (CBC, biochemistry)	X									X
IT infusion (WJ-MSCs or PL)		X			X					
SCIM III	X							X		X
MAS (spasticity)	X							X		X
NRS (neuropathic pain)	X							X		X
EPT, EPPT	X									X
Neurophysiological testing	X									X
Quality of life test (WHOQOL-BREF)	X							X		X
WISCI II Test	X							X		X
Hand muscle strength (dynamometer)	X							X		X
Psychological assessment (PWI, HADS)	X									X
Social worker survey (CIQ-IG)	X									X
Blood sample for patient HLA typing	X									
CSF sample (anti-HLA antibodies)		X	X ^3^	X	X ^3^	X ^3^	X		X ^2^	X ^2^
CSF sample (donor cells)		X	X	X ^3^	X ^3^	X	X ^3^		X ^2^	X ^2^
miRNA biomarkers (blood/CSF) ^1^	X	X	X	X	X	X	X		X ^2^	X ^2^
Rehabilitation programme	X	X	X	X	X	X	X			
Concomitant medication	X	X	X	X	X	X	X	X	X	X
Adverse events	X	X	X	X	X	X	X	X	X	X

^1^ Samples were cryopreserved. ^2^ Only collected if anti-HLA or donor cells were detected. ^3^ Samples were cryopreserved and only analysed if needed. Timepoints/scheduling: mo: month, V: visit, w: week. Interventions: IT: intrathecal, PL: placebo, WJ-MSCs: Wharton’s jelly-derived mesenchymal stromal/stem cells. Clinical scales/outcomes: ASIA: American Spinal Injury Association, CIQ-IG: Community Integration Questionnaire—Institut Guttmann, EPPT: electrical pain perception threshold, EPT: electrical perception threshold, HADS: Hospital Anxiety and Depression Scale, MAS: Modified Ashworth Scale, NRS: numeric rating scale, PWI: Personal Well-Being Index, SCIM III: Spinal Cord Independence Measure III, WHOQOL-BREF: World Health Organization Quality of Life—BREF, WISCI II: Walking Index for Spinal Cord Injury II. Laboratory: CBC: complete blood count, CSF: cerebrospinal fluid, HLA: human leukocyte antigen, miRNA: microRNA.

**Table 3:** Characterisation of WJ-MSC batches.

Attribute	Acceptance Criterion	Average	Standard Deviation
Dose (viable cells/mL)	1 × 10^6^ ± 30%	1.06 × 10^6^	0.06 × 10^6^
Cell viability (%)	≥70	91.2	4.7
Final volume (mL)	4 ± 1	3.7	0.4
CD45^−^/CD105^+^ (%)	≥95	99.0	0.0
CD31^−^/CD173^+^ (%)	≥95	99.7	0.0
CD90^+^ (%)	≥95	99.9	0.0
Sterility ^1^	Negative	Negative	N/A
Endotoxins (EU/mL) ^2^	≤1.00	<1.00	N/A

In total, 20 batches were manufactured and successfully released in conformity with current Good Manufacturing Practice for infusion to SCI patients. ^1^ Sterility testing included adventitious viruses, sterility according to Ph. Eur. 2.6.27, Mycoplasma according to Ph. Eur. 2.6.2. ^2^ Endotoxin testing according to Ph. Eur. 2.6.14. CPD: cumulative population doubling. Italicised values indicate predefined acceptance criteria. N/A: not applicable.

**Table 4:** Demographics, injury characteristics, and randomisation of all patients at baseline.

Patient #	Sex	Age, Years	Weight, kg	Time Since SCI, Years	Neurologic Level	AIS Classification	Randomisation
1	F	59	70	2.1	C4	C	Placebo
2	M	77	82	3.8	C6	C	WJ-MSCs
3	M	46	77	1.1	C5	C	Placebo
4	F	24	49	4.3	C8	B	WJ-MSCs
5	M	29	83	1.5	C6	D	Placebo
6	M	22	83	4.0	C7	C	WJ-MSCs
7	M	22	75	3.3	C7	B	Placebo
8	M	48	85	1.9	C4	C	WJ-MSCs
9	M	44	66	3.3	C8	C	Placebo
10	M	55	74	2.7	C4	D	WJ-MSCs
11	M	37	109	3.2	C2	D	Placebo
12	M	21	75	1.3	C6	B	WJ-MSCs
13	M	21	74	2.6	C5	B	Placebo
14	M	50	80	1.7	C4	D	WJ-MSCs
15	M	34	68	1.1	C4	B	Placebo
16	M	44	95	4.2	C4	C	WJ-MSCs
17	F	49	55	2.9	C4	B	Placebo
18	M	55	79	1.11	C5	D	WJ-MSCs

#: number; AIS: American Spinal Injury Association (ASIA) Impairment Scale; F: female; M: male; SCI: spinal cord injury; WJ-MSCs: Wharton jelly mesenchymal stem cells. Yellow: participant withdrawn from study after first intrathecal infusion (excluded from efficacy analysis; included in safety analysis).

**Table 5:** Motor scores, light touch and pin prick sensation, neurologic level, and AIS classification of participants at baseline and at 12 months.

Patient #	At Baseline					At 12 Months					Randomisation	Completed Study
	Motor Score (R/L)	LT Score (R/L)	PP Score (R/L)	Neurologic Level	AIS	Motor Score (R/L)	LT Score (R/L)	PP Score (R/L)	Neurologic Level	AIS		
1	34/27	33/31	33/33	C4	C	43/31	42/48	34/38	C4	D	Placebo	Yes
3	9/18	14/18	14/17	C5	C	15/28	18/19	16/17	C5	C		Yes
5	28/40	36/56	36/36	C6	D	37/45	35/56	35/56	C6	D		Yes
7	16/14	25/14	12/12	C7	B	18/14	25/14	12/12	C7	B		Yes
9	29/29	35/35	35/35	C8	C	39/38	40/42	38/38	C5	C		Yes
11	42/47	29/30	29/29	C2	D	50/49	56/55	56/44	C4	D		Yes
13	16/22	15/14	14/14	C5	B	16/22	15/14	14/14	C5	B		Yes
15	4/6	16/22	7/6	C4	B	4/7	14/17	7/6	C4	B		Yes
17	8/7	31/30	9/8	C4	B	8/8	30/32	8/6	C4	B		Yes
	Hedges’ g = 1.07 (95% CI 0.25–1.86)/Hedges’ g = 0.90 (95% CI 0.12–1.64)	Hedges’ g = 0.47 (95% CI −0.20–1.12)/Hedges’ g = 0.51 (95% CI −0.17–1.17)	Hedges’ g = 0.37 (95% CI −0.29–1.00)/Hedges’ g = 0.56 (95% CI −0.13–1.23)									
2	25/20	28/28	28/28	C6	C	-	-	-	-	-	WJ-MSCs	No
4	24/24	35/35	35/35	C8	B	25/25	39/39	35/35	C8	B		Yes
6	16/28	37/35	36/35	C7	C	16/29	43/41	36/35	C7	C		Yes
8	10/26	31/31	31/31	C4	C	25/35	41/33	31/33	C4	C		Yes
10	26/50	31/31	31/31	C4	D	39/48	32/56	32/56	C5	D		Yes
12	10/10	34/34	10/10	C6	B	10/10	34/34	10/10	C5	B		Yes
14	24/38	46/50	46/46	C4	D	33/39	56/56	32/32	C5	D		Yes
16	11/23	33/32	7/9	C4	C	14/26	32/31	6/9	C4	C		Yes
18	32/30	51/45	36/19	C5	D	31/32	39/45	28/18	C4	D		Yes
	Hedges’ g = 0.74 (95% CI −0.04–1.48)/Hedges’ g = 0.55 (95% CI −0.18–1.25)	Hedges’ g = 0.30 (95% CI −0.39–0.96)/Hedges’ g = 0.59 (95% CI −0.15–1.29)	Hedges’ g = 0.49 (95% CI −0.23–1.17)/Hedges’ g = 0.13 (95% CI −0.53–0.79)									

#: number; AIS: American Spinal Injury Association (ASIA) Impairment Scale; L: left; LT: light touch; PP: pin prick; R: right; WJ-MSCs: Wharton’s jelly mesenchymal stem cells.

**Table 6:** Main adverse events recorded per patient during 12-month follow-up.

Participant	Group	Adverse Events (AEs)	
		Events	Completed Trial
1	Placebo	No significant AEs	Yes
2	WJ-MSCs	After motor evoked potentials: headache. After the 1st infusion: muscle spasms, pain in lower limbs, back pain, insomnia	No, left study after first infusion due to intolerance of neurophysiological tests. Non-compliance with inclusion criteria (age > 70 years).
3	Placebo	No significant AEs	Yes
4	WJ-MSCs	Post-dural puncture headache requiring hospital admission, autonomic dysreflexia, nausea, urinary tract infection	Yes
5	Placebo	No significant AEs	Yes
6	WJ-MSCs	Post-dural puncture headache, urinary retention with autonomic dysreflexia	Yes
7	Placebo	Post-dural puncture headache	Yes
8	WJ-MSCs	Post-dural puncture headache, urinary retention with autonomic dysreflexia	Yes
9	Placebo	Post-dural puncture headache	Yes
10	WJ-MSCs	No significant AEs	Yes
11	Placebo	Post-dural puncture headache	Yes
12	WJ-MSCs	Post-dural puncture headache, urinary retention, and autonomic dysreflexia	Yes
13	Placebo	Post-dural puncture headache	Yes
14	WJ-MSCs	Post-dural puncture headache	Yes
15	Placebo	Post-dural puncture headache upon rising 5 days after 1st infusion. Headache at 2 days after 2nd infusion	Yes
16	WJ-MSCs	Post-dural puncture headache after 1st infusion. Headache and general malaise after 2nd infusion	Yes
17	Placebo	None after 1st infusion. Post-dural puncture headache after 2nd infusion	Yes
18	WJ-MSCs	Post-dural puncture headache and low-grade fever for 2 days. Mild low back pain after 1st infusion. Headache after 2nd infusion	Yes

**Table 7:** Total motor, light touch (LT), and pin prick (PP) scores at baseline and 12 months.

Outcome	Group	Baseline Total (Mean ± SD) [Median (Q1–Q3)]	12-Month Total (Mean ± SD) [Median (Q1–Q3)]	*p*-Value †
Motor	Placebo (*n* = 9)	44.0 ± 25.0 [38 (21–64.5)]	52.4 ± 29.6 [43 (24–79.5)]	0.009
	WJ-MSCs (*n* = 8)	47.8 ± 17.0 [46 (35–62)]	54.6 ± 19.3 [55 (42.5–67.5)]	0.048
Light touch	Placebo (*n* = 9)	54.6 ± 19.3 [55 (42.5–67.5)]	63.6 ± 29.1 [62 (34–90.5)]	0.152
	WJ-MSCs (*n* = 8)	71.9 ± 13.6 [68 (62–84)]	81.4 ± 14 [81 (71–86)]	0.116
Pin prick	Placebo (*n* = 9)	42.1 ± 22.7 [31 (20.5–68)]	50.1 ± 32.4 [33 (19–83.5)]	0.132
	WJ-MSCs (*n* = 8)	56.0 ± 24.2 [62 (37.5–70.5)]	54.8 ± 24.1 [64 (33–70.5)]	0.818

† paired *t*-test. AIS: American Spinal Injury Association (ASIA) Impairment Scale; Q1–Q3: first–third quartile (interquartile range); SD: standard deviation; WJ-MSCs: Wharton’s jelly-derived mesenchymal stromal/stem cells.

## Data Availability

The data presented in this study are available upon request from the corresponding author due to privacy and ethical restrictions.

## Supplementary Materials

The following supporting information can be downloaded at https://www.mdpi.com/article/10.3390/biomedicines14040762/s1. Table S1: CONSORT 2025 checklist. Table S2: Summary of adverse events during follow-up [41]. Table S3: Distribution of ulnar nerve somatosensory evoked potential (SSEP) results by treatment group. Table S4: Distribution of tibial nerve somatosensory evoked potential (SSEP) results by treatment group. Table S5: Distribution of motor evoked potential (MEP) results for the abductor digiti minimi (ADM) muscle by treatment group. Table S6: Distribution of motor evoked potential (MEP) results for the tibialis anterior (TA) muscle by treatment group. Table S7: Distribution of motor evoked potential (MEP) results for the abductor hallucis (AH) muscle by treatment group.

## Abbreviations

The following abbreviations are used in this manuscript:

ADM	Abductor digiti minimi
AEMPS	Spanish Agency of Medicines and Medical Devices
AH	Abductor hallucis
AIS	ASIA Impairment Scale
ASIA	American Spinal Injury Association
CBC	Complete Blood Count
CFSE	Carboxy-fluorescein diacetate succinimidyl ester
CI	Confidence interval
CIQ-IG	Community Integration Questionnaire—Institut Guttmann Version
CSF	Cerebrospinal fluid
DMEM	Dulbecco’s modified Eagle medium
DP	Drug product
EMG	Electromyography
EP	Evoked potential
EPPT	Electrical pain perception threshold
EPT	Electrical perception threshold
EudraCT	European Union Drug Regulating Authorities Clinical Trials Database
Fmax/Mmax ratio	Maximum F-wave to maximum M-wave ratio
FP	Final product
GMP	Good Manufacturing Practice
H/M ratio	H-reflex to M-wave ratio
HADS	Hospital Anxiety and Depression Scale
HLA	Human leukocyte antigen
H-reflex	Hoffmann reflex
IQR	Interquartile range
ISNCSCI	International Standards for Neurological Classification of Spinal Cord Injury
IT	Intrathecal
MAS	Modified Ashworth Scale
MCB	Master cell bank
MedDRA	Medical Dictionary for Regulatory Activities
MEP(s)	Motor evoked potential(s)
miRNA	microRNA
MRI	Magnetic resonance imaging
MSC(s)	Mesenchymal stromal/stem cells
NRS	Numerical rating scale
PBMC	Peripheral blood mononuclear cells
PWI	Personal Well-Being Index
QST	Quantitative sensory testing
RCT	Randomised controlled trial
SCI	Spinal cord injury
SCIM III	Spinal Cord Independence Measure, Version III
SD	Standard deviation
SOC	System organ class
SSEP(s)	Somatosensory evoked potential(s)
TA	Tibialis anterior
TMS	Transcranial magnetic stimulation
WISCI II	Walking Index for Spinal Cord Injury, Version II
WJ-MSC(s)	Wharton’s jelly-derived mesenchymal stromal/stem cell(s)
WHOQOL-BREF	World Health Organization Quality of Life—BREF
