# Supplementary material for: Mesenchymal Stromal/Stem Cells in Chronic Incomplete Traumatic Spinal Cord Injury: A Phase I/II Double-Blind Placebo-Controlled Multicentre Trial
Source: Biomedicines. 2026 Mar 26;14(4):762. doi: 10.3390/biomedicines14040762 (PMC13113423; doi:10.3390/biomedicines14040762)
Supplement: Supplementary file 1 [file biomedicines-14-00762-s001.zip › biomedicines-4141959-supplementary.pdf]

Table S1. CONSORT 2025 checklist.

Section / Topic	No	CONSORT 2025 checklist item description	Reported on page no.
**Title and abstract**			
Title and structured abstract	1a	Identification as a randomised trial	1
	1b	Structured summary of the trial design, methods, results, and conclusions	1
**Open science**			
Trial registration	2	Name of trial registry, identifying number (with URL) and date of registration	35
Protocol and statistical analysis plan	3	Where the trial protocol and statistical analysis plan can be accessed	7 (Protocol); 10 (Statistical analysis plan)
Data sharing	4	Where and how the individual de-identified participant data (including data dictionary), statistical code and any other materials can be accessed	35
Funding and conflicts of interest	5a	Sources of funding and other support (e.g., supply of drugs), and role of funders in the design, conduct, analysis and reporting of the trial	35
	5b	Financial and other conflicts of interest of the manuscript authors	35
**Introduction**			
Background and rationale	6	Scientific background and rationale	2
Objectives	7	Specific objectives related to benefits and harms	2
**Methods**			
Patient and public involvement	8	Details of patient or public involvement in the design, conduct and reporting of the trial	Patients and public were not involved in study designing.
Trial design	9	Description of trial design including type of trial (e.g., parallel group, crossover), allocation ratio, and framework (e.g., superiority, equivalence, non-inferiority, exploratory)	3
Changes to trial protocol	10	Important changes to the trial after it commenced including any outcomes or analyses that were not prespecified, with reason	No important changes were made.
Trial setting	11	Settings (e.g., community, hospital) and locations (e.g., countries, sites) where the trial was conducted	5
Eligibility criteria	12a	Eligibility criteria for participants	3
	12b	If applicable, eligibility criteria for sites and for individuals delivering the interventions (e.g., surgeons, physiotherapists)	Not applicable
Intervention and comparator	13	Intervention and comparator with sufficient details to allow replication. If relevant, where additional materials describing the intervention and comparator (e.g., intervention manual) can be accessed	3-6
Outcomes	14	Pre-specified primary and secondary outcomes, including the specific measurement variable (e.g., systolic blood pressure), analysis metric (e.g., change from baseline, final value, time to event), method of aggregation (e.g., median, proportion), and time point for each outcome	8-10
Harms	15	How harms were defined and assessed (e.g., systematically, non-systematically)	10

Sample size	16a	How sample size was determined, including all assumptions supporting the sample size calculation	3
	16b	Explanation of any interim analyses and stopping guidelines	Not applicable
Randomisation:			
Sequence generation	17a	Who generated the random allocation sequence and the method used	3
	17b	Type of randomisation and details of any restriction (e.g., stratification, blocking and block size)	3
Allocation concealment mechanism	18	Mechanism used to implement the random allocation sequence (e.g., central computer/telephone; sequentially numbered, opaque, sealed containers), describing any steps to conceal the sequence until interventions were assigned	3
Implementation	19	Whether the personnel who enrolled and those who assigned participants to the interventions had access to the random allocation sequence	3
Blinding	20a	Who was blinded after assignment to interventions (e.g., participants, care providers, outcome assessors, data analysts)	3
	20b	If blinded, how blinding was achieved and description of the similarity of interventions	3
Statistical methods	21a	Statistical methods used to compare groups for primary and secondary outcomes, including harms	10
	21b	Definition of who is included in each analysis (e.g., all randomised participants), and in which group	10
	21c	How missing data were handled in the analysis	10
	21d	Methods for any additional analyses (e.g., subgroup and sensitivity analyses), distinguishing prespecified from post-hoc	10
**Results**			
Participant flow, including flow diagram	22a	For each group, the numbers of participants who were randomly assigned, received intended intervention, and were analysed for the primary outcome	3-4 (in Methods)
	22b	For each group, losses and exclusions after randomisation, together with reasons	3-4 (in Methods)
Recruitment	23a	Dates defining the periods of recruitment and follow-up for outcomes of benefits and harms	5 (in Methods)
	23b	If relevant, why the trial ended or was stopped	5 (in Methods)
Intervention and comparator delivery	24a	Intervention and comparator as they were actually administered (e.g., where appropriate, who delivered the intervention/comparator, how participants adhered, whether they were delivered as intended [fidelity])	14-15
	24b	Concomitant care received during the trial for each group	5 (in Methods)
Baseline data	25	A table showing baseline demographic and clinical characteristics for each group	11
Numbers analysed, outcomes and estimation	26	For each primary and secondary outcome, by group:<br>● the number of participants included in the analysis<br>● the number of participants with available data at the outcome time point<br>● result for each group, and the estimated effect size and its precision (such as 95% confidence interval)<br>● for binary outcomes, presentation of both absolute and relative effect size	11-18
Harms	27	All harms or unintended events in each group	15, 26
Ancillary analyses	28	Any other analyses performed, including subgroup and sensitivity analyses, distinguishing pre-specified from post-hoc	14-18
**Discussion**			
Interpretation	29	Interpretation consistent with results, balancing benefits and harms, and considering other relevant evidence	18-20
Limitations	30	Trial limitations, addressing sources of potential bias, imprecision, generalisability, and, if relevant, multiplicity of analyses	20

*We strongly recommend reading this statement in conjunction with the CONSORT 2025 Explanation and Elaboration and/or the CONSORT 2025 Expanded Checklist for important clarifications on all the items. We also recommend reading relevant CONSORT extensions. See www.consort-spirit.org.

Citation: Hopewell S, Chan AW, Collins GS, Hróbjartsson A, Moher D, Schulz KF, et al. CONSORT 2025 Statement: updated guideline for reporting randomised trials. BMJ. 2025; 388:e081123. https://dx.doi.org/10.1136/bmj-2024-081123.

© 2025 Hopewell et al. This is an Open Access article distributed under the terms of the Creative Commons Attribution License (https://creativecommons.org/licenses/by/4.0/), which permits unrestricted use, distribution, and reproduction in any medium, provided the original work is properly cited.

Table S2. Summary of adverse events during follow-up.

(A)

**Metric**	**Placebo (n=9)**	**WJ-MSC (n=9)**	**Total (n=18)**
Participants with ≥1 AE, n (%)	9 (100%)	9 (100%)	18 (100%)
Total AEs, n	26	54	80
Events per participant (events/n)	2.89	6.00	4.44
Participants with ≥1 SAE, n (%)	1 (11.1%)	5 (55.6%)	6 (33.3%)
Total SAE, n	1	6	7
Participants with ≥1 treatment-related AE, n (%)	2 (22.2%)	3 (33.3%)	5 (27.8%)
Treatment-related AEs, n	2	6	8

(B)

**AE intensity**	**Placebo**	**WJ-MSC**	**Total**
Mild	20 events; 8 participants	32 events; 8 participants	52 events; 16 participants
Moderate	5 events; 5 participants	19 events; 8 participants	24 events; 13 participants
Severe	1 event; 1 participant	3 events; 2 participants	4 events; 3 participants

(C)

**SOC**	**Placebo**	**WJ-MSC**	**Total**
Nervous system disorders	17; 8	21; 9	38; 17
Musculoskeletal & connective tissue disorders	2; 2	14; 4	16; 6
Infections & infestations	2; 1	8; 3	10; 4
Gastrointestinal disorders	1; 1	4; 2	5; 3
Vascular disorders	1; 1	3; 3	4; 4
General disorders & administration site conditions	0; 0	2; 2	2; 2
Surgical & medical procedures	2; 2	0; 0	2; 2
Accidents & injuries	0; 0	1; 1	1; 1
Psychiatric disorders	1; 1	0; 0	1; 1
Renal & urinary disorders	0; 0	1; 1	1; 1

(A) Number of adverse events (AEs) and number of serious adverse events (SAEs) per group; (B) Classification of AEs per treatment group according to AE intensity; (C) Classification of AEs per treatment group as per MedDRA System Organ Class (SOC) (events; participants affected). SOC: MedDRA System Organ Class. Values are presented as number of events and number (%) of participants affected, if applicable.

Table S3. Distribution of ulnar nerve somatosensory evoked potentials (SSEP) results by treatment group.

		**Ulnar nerve SSEP at baseline**						**Ulnar nerve SSEP at 12 months**					
**Patient**	**Treatment Group**	**Lat R (ms)**	**Lat R (ms)**	**Amp R (μV)**	**Lat L (ms)**	**Lat L (ms)**	**Amp L (μV)**	**Lat R (ms)**	**Lat R (ms)**	**Amp R (μV)**	**Lat L (ms)**	**Lat L (ms)**	**Amp L (μV)**
		N19	P22		N19	P22		N19	P22		N19	P22	
1	placebo	23.7	29.85	2.4	-	-	0	20.3	23.4	1.4	-	-	0
3	placebo	-	-	0	-	-	0	-	-	0	-	-	0
5	placebo	21.9	24.9	1.8	21	23.2	1.6	21.3	24.8	1.1	21.1	24	1.2
7	placebo	-	-	0	-	-	0	-	-	0	-	-	0
9	placebo	20.7	22.95	7.8	21	24	3.3	22.5	24.6	2	21.3	24.4	2.6
11	placebo	21.1	24.8	1.5	22.3	25.6	1.1	20.7	25.2	1.8	21.8	24.8	1.4
13	placebo	22.2	26.1	1.4	22	26.2	1.6	21.7	25.8	1	22.3	26.7	1.3
15	placebo	-	-	0	-	-	0	-	-	0	-	-	0
17	placebo	-	-	0	-	-	0	-	-	0	-	-	0
p-value		0.44 Hedges' g = 0.30 (95% CI -1.10 – 0.53)	1.00 Hedges' g = 0.27 (95% CI -1.07 – 0.55)	0.13 Hedges' g = 0.42 (95% CI -1.07 – 0.24)	0.88 Hedges' g = 0.12 (95% CI -0.75 – 0.96)	0.75 Hedges' g = 0.28 (95% CI -0.62 – 1.13)	0.38 Hedges' g = 0.40 (95% CI -1.03 – 0.27)						
2	WJ-MSC	-	-	0	-	-	0	*participant withdrawn from study*					
4	WJ-MSC	19.9	23.4	2.7	19.9	23.1	2.2	18.9	-	3	20.2	-	2
6	WJ-MSC	-	-	0	0	-	0	22.9	26.5	2.9	21.7	24.7	4.9
8	WJ-MSC	-	-	0	-	-	0	-	-	0	19.1	21	0.5
10	WJ-MSC	22.95	27.3	0.71	24.8	28.6	0.72	21.7	27.1	2	24.9	27.4	0.79
12	WJ-MSC	22.75	25.6	0.73	-	-	0	-	-	0	-	-	0
14	WJ-MSC	25.4	29.3	1.2	27.3	33.1	1	26.2	28	1.2	23.1	25	0.98
16	WJ-MSC	-	-	0	-	-	0	-	-	0	-	-	0
18	WJ-MSC	-	-	0	-	-	0	-	-	0	-	-	0
p-value		0.50 Hedges' g = 0.35 (95% CI -1.27 – 0.65)	0.50 Hedges' g = 0.54 (95% CI -1.51 – 0.52)	0.38 Hedges' g = 0.39 (95% CI -0.31 – 1.07)	0.63 Hedges' g = 0.33 (95% CI -0.58 – 1.20)	0.50 Hedges' g = 0.54 (95% CI -1.50 – 0.52)	0.44 Hedges' g = 0.36 (95% CI -0.33 – 1.03)						

p-values were calculated using the Wilcoxon test (two-tailed). Amp: amplitude; Lat: latency; L: left; R: right; ms: milliseconds; μV: microvolts; N19: N19 peak; P22: P22 peak; SSEP: somatosensory evoked potential; WJ-MSC: Wharton's jelly-derived mesenchymal stromal/stem cells.

Table S4. Distribution of tibial nerve somatosensory evoked potentials (SSEP) results by treatment group.

		**Tibial nerve SSEP at baseline**						**Tibial nerve SSEP at 12 months**					
**Patient**	**Treatment Group**	**Lat R (ms)**	**Lat R (ms)**	**Amp R (μV)**	**Lat L (ms)**	**Lat L (ms)**	**Amp L (μV)**	**Lat R (ms)**	**Lat R (ms)**	**Amp R (μV)**	**Lat L (ms)**	**Lat L (ms)**	**Amp L (μV)**
		N40	P50		N40	P50		N40	P50		N40	P50	
1	placebo	-	-	0	-	-	0	-	-	0	-	-	0
3	placebo	-	-	0	-	-	0	-	-	0	-	-	0
5	placebo	41.7	48.6	1.1	42.4	47.2	0.8	42.1	50.5	0.94	42.3	48.9	0.87
7	placebo	-	-	0	-	-	0	-	-	0	-	-	0
9	placebo	-	-	0	-	-	0	50.7	55.6	0.7	50.1	67.5	1.6
11	placebo	46.9	53.9	1.8	45.7	53.4	1.4	43.3	51.4	1.6	42.2	50	1.6
13	placebo	-	-	0	-	-	0	-	-	0	-	-	0
15	placebo	-	-	0	-	-	0	-	-	0	-	-	0
17	placebo	-	-	0	-	-	0	-	-	0	-	-	0
p-value		1.00	1.00	1.00	0.50	1.00	0.25						
		Hedges' g = 0.32 (95% CI -1.14 – 0.60)	Hedges' g = 0.05 (95% CI -0.83 – 0.74)	Hedges' g = 0.14 (95% CI -0.49 – 0.76)	Hedges' g = 0.42 (95% CI -1.30 – 0.56)	Hedges' g = 0.13 (95% CI -0.91 – 0.69)	Hedges' g = 0.38 (95% CI -0.28 – 1.01)						
2	WJ-MSC	-	-	0	-	-	0	*participant withdrawn from study*					
4	WJ-MSC	-	-	0	-	-	0	-	-	0	-	-	0
6	WJ-MSC	41.8	52.5	4.2	45.1	53.2	1.5	53.7	63.4	2.7	53.4	66.4	1.6
8	WJ-MSC	-	-	0	-	-	0	-	-	0	-	-	0
10	WJ-MSC	41.3	49.6	0.73	43.4	49.6	0.78	40.6	46.5	0.91	42.1	50.2	1.6
12	WJ-MSC	-	-	0	65.25	75.15	0.56	-	-	0	-	-	0
14	WJ-MSC	-	-	0	40.3	49.2	1	43.3	49.4	0.7	47.6	51.9	0.89
16	WJ-MSC	-	-	0	-	-	0	-	-	0	-	-	0
18	WJ-MSC	-	-	0	-	-	0	-	-	0	-	-	0
p-value		1.00	0.50	1.00	0.50	0.25	1.00						
		Hedges' g = 0.36 (95% CI -0.58 – 1.19)	Hedges' g = 0.22 (95% CI -0.64 – 1.01)	Hedges' g = 0.12 (95% CI -0.77 – 0.54)	Hedges' g = 0.72 (95% CI -0.44 – 1.79)	Hedges' g = 0.65 (95% CI -0.48 – 1.68)	Hedges' g = 0.08 (95% CI -0.58 – 0.73)						

p-values were calculated using the Wilcoxon test (two-tailed). Amp: amplitude; Lat: latency; L: left; ms: milliseconds; μV: microvolts; N40: N40 peak; P50: P50 peak; R: right; WJ-MSC: Wharton's jelly-derived mesenchymal stromal/stem cells.

Table S5. Distribution of motor evoked potentials (MEP) results for the abductor digiti minimi (ADM) muscle by treatment group.

		**ADM MEP at baseline**				**ADM MEP at 12 months**			
**Patient**	**Treatment Group**	**Lat R (ms)**	**Amp R (μV)**	**Lat L (ms)**	**Amp L (μV)**	**Lat R (ms)**	**Amp R (μV)**	**Lat L (ms)**	**Amp L (μV)**
1	placebo	-	0	-	0	25.6	842	24.9	744
3	placebo	45.6	205	32.7	262	-	0	19.9	151
5	placebo	28.4	93	27.1	185	25	517	24.1	329
7	placebo	-	0	-	0	-	0	-	0
9	placebo	22.3	61.7	22.8	2000	20	5290	21.7	2750
11	placebo	23.7	1304.9	23.7	963.4	23.4	1634	23.3	743.9
13	placebo	28.4	202	25.3	994	28.1	500	25.8	1600
15	placebo	-	0	-	0	-	0	-	0
17	placebo	-	0	-	0	-	0	-	0
p-value		0.13 Hedges' g = 0.89 (95% CI -1.94 – 0.24)	0.06 Hedges' g = 0.43 (95% CI -0.24 – 1.07)	0.19 Hedges' g = 0.56 (95% CI -1.41 – 0.34)	0.22 Hedges' g = 0.53 (95% CI -0.16 – 1.19)				
2	WJ-MSC	27.7	335	29.6	283	*participant withdrawn from study*			
4	WJ-MSC	21	2695	21.7	4415	18.5	3612	20.4	6305
6	WJ-MSC	-	0	30	451	-	0	27.9	451
8	WJ-MSC	-	0	-	0	-	0	28.3	242
10	WJ-MSC	26.3	146	27.2	349	27	98.8	25.7	165.9
12	WJ-MSC	-	0	-	0	-	0	-	0
14	WJ-MSC	31.8	110	33.9	104	-	0	-	0
16	WJ-MSC	-	0	-	0	-	0	-	0
18	WJ-MSC	-	0	-	0	-	0	-	0
p-value		1.00 Hedges' g = 0.22 (95% CI -1.01 – 0.64)	1.00 Hedges' g = 0.27 (95% CI -0.41 – 0.93)	0.25 Hedges' g = 3.13 (95% CI 0.28 – 6.12)	0.63 Hedges' g = 0.32 (95% CI -0.37 – 0.99)				

p-values were calculated using the Wilcoxon test (two-tailed). Amp: amplitude; Lat: latency; L: left; ms: milliseconds; μV: microvolts; R: right; WJ-MSC: Wharton's jelly-derived mesenchymal stromal/stem cells.

Table S6. Distribution of motor evoked potentials (MEP) results for the tibialis anterior (TA) muscle by treatment group.

		**TA MEP at baseline**				**TA MEP at 12 months**			
**Patient**	**Treatment Group**	**Lat R (ms)**	**Amp R (μV)**	**Lat L (ms)**	**Amp L (μV)**	**Lat R (ms)**	**Amp R (μV)**	**Lat L (ms)**	**Amp L (μV)**
1	placebo	-	0	-	0	-		-	0
3	placebo	-	0	-	0	-	0	-	0
5	placebo	-	0	44.7	945	-	0	26.7	918
7	placebo	-	0	-	0	-	0	-	0
9	placebo	43	110	47.9	585	46	396	50.3	726
11	placebo	32.3	746	31.7	1043	32.2	937	33.4	812
13	placebo	-	0	-	0	-	0	-	0
15	placebo	-	0	-	0	-	0	-	0
17	placebo	-	0	-	0	-	0	-	0
p-value		1.00<br>Hedges' g = 0.37 (95% CI -0.58 – 1.22)	0.50<br>Hedges' g = 0.50 (95% CI -0.22 – 1.19)	1.00<br>Hedges' g = 0.32 (95% CI -1.24 – 0.66)	0.75<br>Hedges' g = 0.13 (95% CI -0.75 – 0.50)				
2	WJ-MSC	55.9	90	54.7	30	*participant withdrawn from study*			
4	WJ-MSC	-	0	-	0	18.5	3612	20.4	6305
6	WJ-MSC	-	0	-	0	-	0	27.9	451
8	WJ-MSC	-	0	-	0	-	0	28.3	242
10	WJ-MSC	-	0	36.6	128	27	98.8	25.7	165.9
12	WJ-MSC	-	0	-	0	-	0	-	0
14	WJ-MSC	-	0	-	0	-	0	-	0
16	WJ-MSC	-	0	-	0	-	0	-	0
18	WJ-MSC	-	0	-	0	-	0	-	0
p-value		N/A	N/A	N/A	1.00<br>Hedges' g = 0.32 (95% CI -0.37 – 0.99)				

N/A: not available. *p*-values were calculated using the Wilcoxon test (two-tailed). Amp: amplitude; Lat: latency; L: left; ms: milliseconds; μV: microvolts; R: right; WJ-MSC: Wharton's jelly-derived mesenchymal stromal/stem cells.

Table S7. Distribution of motor evoked potentials (MEP) results for the abductor hallucis (AH) muscle by treatment group.

		**AH MEP at baseline**				**AH MEP at 12 months**			
**Patient**	**Treatment Group**	**Lat R (ms)**	**Amp R (μV)**	**Lat L (ms)**	**Amp L (μV)**	**Lat R (ms)**	**Amp R (μV)**	**Lat L (ms)**	**Amp L (ms)**
1	placebo	-	0	-	0	-	0	-	0
3	placebo	-	0	50.3	149	-	0	44.5	198
5	placebo	55.2	122	50	250	44.1	363	47.7	302
7	placebo	-	0	-	0	-	0	-	0
9	placebo	58.7	773	53	1939	54.3	518	50.7	1100
11	placebo	46.9	317	46.2	561	46	846	43.8	317
13	placebo	-	0	-	0	-	0	-	0
15	placebo	-	0	-	0	-	0	-	0
17	placebo	-	0	-	0	-	0	-	0
p-value		0.25<br>Hedges' g = 0.84 (95% CI -1.98 – 0.39)	0.75<br>Hedges' g = 0.25 (95% CI -0.39 – 0.88)	0.13<br>Hedges' g = 1.60 (95% CI 0.09 – 3.06)	0.63<br>Hedges' g = 0.36 (95% CI -1.00 – 0.30)				
2	WJ-MSC	60.5	242	52.9	128	*participant withdrawn from study*			
4	WJ-MSC	-	0	-	0	-	0	-	0
6	WJ-MSC	-	0	-	0	-	0	-	0
8	WJ-MSC	-	0	-	0	-	0	57.3	168
10	WJ-MSC	46.1	66	42.3	1542.7	42.9	56.1	38.4	1171
12	WJ-MSC	-	0	-	0	-	0	-	0
14	WJ-MSC	51.9	354	47	829	-	0	-	0
16	WJ-MSC	-	0	-	0	-	0	-	0
18	WJ-MSC	-	0	-	0	-	0	-	0
p-value		N/A	0.50<br>Hedges' g = 0.35 (95% CI -1.01 – 0.35)	N/A	0.50<br>Hedges' g = 0.38 (95% CI -1.05 – 0.32)				

N/A: not available. *p*-values were calculated using the Wilcoxon test (two-tailed). Amp: amplitude; Lat: latency; L: left; ms: milliseconds; μV: microvolts; R: right; WJ-MSC: Wharton's jelly-derived mesenchymal stromal/stem cells.
